# Beyond the Words: Comparing Interpersonal Engagement Between Maternal and Paternal Infant-Directed Speech Acts

**DOI:** 10.3389/fpsyg.2020.523551

**Published:** 2020-12-03

**Authors:** Theano Kokkinaki, Vassilis G. S. Vasdekis

**Affiliations:** ^1^Laboratory of Applied Psychology, Department of Psychology, University of Crete, Rethymnon, Greece; ^2^Department of Statistics, Athens University of Economics and Business, Athens, Greece

**Keywords:** questions, direct requests, facial expressions, emotions, interpersonal engagement, innate intersubjectivity, infancy

## Abstract

The present study investigates the way infants express their emotions in relation to parental feelings between maternal and paternal questions and direct requests. We therefore compared interpersonal engagement accompanying parental questions and direct requests between infant–mother and infant–father interactions. We video-recorded spontaneous communication between 11 infant–mother and 11 infant–father dyads—from the 2nd to the 6th month—in their home. The main results of this study are summarized as follows: (a) there are *similarities* in the way preverbal infants use their affections in spontaneous interactions with their mothers and fathers to express signs of sensitivity in sharing knowledge through questions and direct requests; and (b) the developmental trajectories of face-to-face emotional coordination in the course of parental questions descend in a similar way for both parents across the age range of this study. Regarding the developmental trajectories of emotional non-coordination, there is evidence of a linear trend in terms of age difference between the parents’ gender with fathers showing the steeper slope. The results are discussed in relation to the theory of intersubjectivity.

## Introduction

Young infants are born with motives that lead them to communicate their feelings with caregivers in order to have their needs met (Trevarthen, 1993a,b, 2011, 2012, 2017). Before language, an infant is considered a person who can participate with an affectionate partner through movements that express feelings for the purpose of negotiation and sharing of meaning (Bullowa, 1979; Reissland, 2012). Young infants (before the 6th month) ‘*read’* the intentions inherent in their parents’ actions (Trevarthen et al., 2006). Parents, both mothers and fathers, are sensitive to the infant’s capacities for emotional regulation, coordination, and communication of feelings. In this way, parents build a unique relationship of intimacy with their infant (Trevarthen et al., 2006).

Evidence on young infants’ sensitivity to sentence-type discrimination comes mainly from the field of speech analysis. Within this field, research on infant’s sensitivity to sentence-type distinction through observations of spontaneous infant–parent interactions is rare and even more seldom when comparing mothers and fathers.

### Infant Speech Perception Development: Why Is Infant Sensitivity in Discrimination of Speech Acts Still an Open Matter?

The foundation for speech perception and language acquisition is laid before birth. In the last months of pregnancy, fetuses are capable of detecting, recognizing, responding to, and remembering some characteristics of the maternal voice (Kisilevsky et al., 2003; Voegtline et al., 2013). Prenatal exposure to maternal voice and heartbeat sounds may constitute the neural pathways for auditory entrainment and synchrony skills necessary for interpersonal communication (Kisilevsky et al., 2003). In addition, fetuses encode some suprasegmental characteristics of speech (intonation and rhythm) into memory (DeCasper and Spence, 1986). When fetal responses to the recorded maternal and paternal voices were compared, contrasting evidence showed the following: (a) that an increase in the heart was observed during exposure to the mother’s but not father’s voice (following the offset of the father’s voice, fetuses responded with a brief heart rate increase) (Kisilevsky et al., 2009; Lee and Kisilevsky, 2013); and (b) fetuses responded in a similar manner to both maternal and paternal voices before birth. After birth, neonates showed a preference for the mother’s voice and no preference for the father’s voice (Lee and Kisilevsky, 2013).

Regarding infant speech discrimination, there is contradictory evidence on young infants’ ability to discriminate consonant contrasts (Eimas et al., 1971; Eimas, 1974; Lasky et al., 1975; Eimas and Miller, 1980) and vowels (Trehub, 1973; Swoboda et al., 1976; Kuhl, 1983). Recent evidence may favor the proposition that infants come pre-wired with auditory processing skills that are modified selectively by experience in the language-learning environment (Houston, 2016). Findings on word segmentation suggest that well before infants are able to produce words, by 8 months of age, they are able to segment words from fluent speech (Jusczyk and Aslin, 1995). Young infants’ sensitivity to prosodic patterns of their native language (as early as 2 months) constitutes one of the potential sources of information that may facilitate infant word segmentation ability (for a full discussion see Houston, 2005).

In connection with the evidence on fetal suprasegmental information encoding, there is evidence that, after birth, 4-month-old infants’ greater attention to infant-directed speech compared to adult-directed speech has been attributed to positive affect conveyed. Positive affect of infant-directed speech is carried through certain aspects of pitch, particularly through intonation (Fernald and Kuhl, 1987; Fernald, 1989, 1993).

Linguistically focused analyses for the examination of infant discrimination of sentence types start as early as 5 months, and it has been mainly based on visual habituation paradigm. There is evidence that infants are specifically able to discriminate the intonational characteristics of questions from those of statements and exclamations and prefer to listen to questions over statements (Best et al., 1991; Geffen and Mintz, 2011; Soderstrom et al., 2011; Frota et al., 2014). In the meantime, certain methodological issues of these studies imply that young infants’ sentence-type distinctions, according to a straightforward relationship with prosody, remains an open matter (Van de Weijer, 2002; Geffen and Mintz, 2017; Reimchen and Soderstrom, 2017). What is more, discrimination of, and preference for, the intonational forms of questions, does not show whether infants treat questions as having a different communicative function from declaratives. On this ground, taking into account the infants’ active participation in ‘dialogues’ with their mothers may constitute a new perspective in the research on infant sentence-type discrimination (Reimchen and Soderstrom, 2017).

### Previous Studies Comparing Emotional Engagement and Speech Acts Between Early Mother–Infant and Father–Infant Playful Interactions

Several studies have compared emotional engagement in playful interactions of infants with their mother and father in the first semester of life. Some researchers did not find significant differences in parental attempts to direct infant’s attention, sensitivity and synchrony (Landerholm and Scriven, 1981; Braungart-Rieker et al., 1998; Feldman, 2003; Branger et al., 2019). O thers described variations between mothers and fathers in playful behavior, responsiveness, emotional matching, and attunement (Yogman, 1981; Belsky et al., 1984; Forbes et al., 2004; Kokkinaki and Vasdekis, 2015).

A limited number of studies have shown variations in emotional expressions in interactions of infants with their mothers and fathers. Infants respond more positively to mothers than to fathers. Mothers express more positive but also more negative affect than fathers (Forbes et al., 2004; Jia, 2014). In the meanwhile, compared to mothers, fathers express more interest and less neutral affect toward their infant (Jia, 2014; Cerezo et al., 2017).

Speech acts addressed by parents to young infants are components of parent–infant communication. Studies focus mainly on the verbal design of this communication, such as frequency and types of questions. Parental infant-directed questions did not differ significantly between mothers and fathers while mothers made more requests than fathers (Kruper and Uzgiris, 1987; Laflamme et al., 2002; Kokkinaki, 2018).

However, the conclusion is based on different family studies varying in their methodology. They lack investigating the early developmental path through which preverbal young infants show sensitivity to the intentions inherent in parents’ different sentence-types. Further, they fail to take into account the inter-sensory nature of communication, i.e., both speech acts and facial expressions of emotion.

### Hypotheses and Importance of This Study

Based on the theory of intersubjectivity (Trevarthen et al., 2006), both parents, the mother and the father, are sensitive communicative partners to the infants and they build a unique relationship of intimacy with them. Young infants ‘*read’* the intentions inherent in their parents’ actions by coordinating their expressions and forming the basis of embodied intersubjectivity (Trevarthen et al., 2006). In the first semester of life, there are age-related transformations in the way infants regulate their feelings and express their interests to their companions (Trevarthen and Aitken, 2003). In the same period, relevant studies comparing emotional engagement in playful interactions of infants with their mother and father did not find significant differences in parental sensitivity and synchrony, but they showed variations in emotional matching and attunement (Braungart-Rieker et al., 1998; Feldman, 2003; Kokkinaki and Vasdekis, 2015; Branger et al., 2019).

Based on this, we addressed the following *hypotheses*.

*Hypothesis 1*: When mothers and fathers ask questions (Hypothesis 1a) and make directive signals (Hypothesis 1b) to their infants, we expect that infants will be similar in the way they will synchronize the shifts of their emotional states between mothers and fathers (synchrony). Further, we expect that mothers and fathers will be similar in the way they will respond with sensitivity to infant negative and neutral emotional expressions. In the meanwhile, we anticipate variations in the way infants will match pleasure and interest facial expressions to the same states of mothers and fathers (matching), and they will complete their mothers’ and fathers’ emotional expression of positive valence (pleasure or interest) (completion). In addition, we expect that infants’ emotional intensity will be differentially affected by the corresponding expression of emotional intensity by the mother and the father (attunement).

*Hypothesis 2*: Infant age will significantly affect, and in a way that is similar between the two dyads, the interpersonal engagement (coordination/non-coordination) taking place during maternal and paternal questions.

This study is important because we know very little about how young infants (before the 6th month) ‘*read’* the intentions inherent in their parents’ actions (Trevarthen et al., 2006). Furthermore, the vocal and verbal experiences to which infants are exposed cannot be not separately considered from facial expressions accompanying them (Jaffe et al., 1973). Within this context, this study may extend our understanding of the intersubjective function of parental infant-directed questions and direct requests (Sylvester-Bradley and Trevarthen, 1978; Trevarthen, 1997; Reddy, 2015; Kokkinaki, 2017). In the meanwhile, male and female speakers may differ in the way they convey the same affective messages to their infants (Slaney and McRoberts, 2003). The ‘*emotion lessons*,’ combining facial/vocal expressions, available during mother–child and father–child interactions may have significant effects on infants’ developing understanding of others’ emotion expressions (Montague and Walker-Andrews, 2002).

## Materials and Methods

### Participants

Participants were part of a cross-cultural and longitudinal study which investigated emotional expressions during the course of imitations in dyadic interactions of infants with their mothers and fathers coming from Greek and Scottish families (*N* = 90 subjects) (see Note). Ethical approval of the project was given by the Lothian Research Ethics Committee and the Psychiatry/Clinical Psychology Research Ethics Sub-Committee of the Royal Infirmary of Edinburgh (NHS Trust).

For the current investigation, 11 mother–infant pairs and 11 father–infant pairs from 11 families were taken randomly from the Greek sample (*N* = 33). All parents were native Greek speakers. Infants with complications at birth, or with known abnormality of development, as well as single parent families and those with children from previous relationships and with health or drug problems were excluded from the sample of this study. Families did not receive compensation for participating in the study. Table 1 presents demographic information for the sample of this study.

**TABLE 1:** Demographic information for the sample.

	Mean	*SD*	Range
Mother Age (years)	29.36	5.12	21–39
Mother Education (years)	14.36	2.33	12–18
Father Age (years)	33.63	5.69	27–47
Father Education (years)	14.36	2.33	12–18
Birth Weight (kg)	3.568	487.47	2,800–4,250
Birth Height (cm)	52.22	2,160	48–55
Breastfeeding (days)	77.71	74.40	14–180
Male/Female	5 (45.5%) male and 6 (54.5%) female		
Way of Delivery	8 (72.7) vaginal deliveries and 3 (27.3%) caesarian sections		
Birth Order	7 (63.7%) first-born and 4 (36.3%) second-born		
Family Composition	Two-parent families		
Paternal presence at labor and birth	36.4%		
Caretaking	Mother was the primary caretaker and father was the secondary caretaker for 4 infants (36.4%). For the remaining 7 infants (63.6%) other persons (such as grandmother, grandfathers, aunt and nanny) took care of them along with their parents		
Marital Status	Married		
Socio-economic Status	Middle-class families		

### Procedure

After parents had chosen to participate in the study (see Kokkinaki, 1998 for details on subject recruitment), an introductory discussion with the researcher took place at their home. Parents were informed about the procedure of the study and asked to sign the consent form prior to video recording. Visits at infants’ home and video recordings were scheduled at a time when the infants were reported by their mothers to be alert and playful and when both the mother and the father were at home. According to video recording schedule, the researcher visited infants’ home and video recorded dyadic interactions of infants with their mother and father at 15-day intervals. Video recordings started at the end of the Primary Intersubjectivity Period (2 months) and continued until the end of Period of Games I (6 months) (Trevarthen, 1984).

During each visit, the order of video recordings of infants interacting with their parents was counterbalanced. In the first visit, video recordings always began with the parent of the same sex as the infant and the order switched in each successive visit. The instruction given to the parents was “Play as you normally do with your baby.”

Given that infant’s state and age constitute factors that appear to influence arousal, attention and affect (Field, 1981), we varied the duration of video-recordings across the age range of this study. Each video-recording lasted 8 min for the younger infants, aged 2–4 months, and 10 min for the older infants, aged 4.5–6 months.

A total of 198 recordings were made for the whole sample. The total duration of micro-analyzed interactions was 1760 min, equally distributed between interactions with mother and father and without any data loss (see Kokkinaki et al., 2016, for further information on the recording conditions).

### Coding

#### Plan of Micro-Analysis and Coding

Micro-analysis of spontaneous infant and parent facial expressions of emotion during parental questions and direct requests was carried out according to the following procedure:

(1) maternal and paternal infant-directed speech and infant vocalizations/non-speech sounds were transcribed from the infant–parent video recordings by the researcher, and transcripts were then checked for accuracy by an assistant;

(2) transcripts of parental speech addressed to the infants were classified into *‘content categories,’ ‘thematic sequences,’* into *‘focus categories’*;

(3) each focus category and the thematic sequences were grouped into units and subunits of analysis, and this was done on the basis of the pause duration preceding and following each thematic sequence;

(4) within each subunit of analysis, each focus category and within thematic sequences we coded for (a) speech acts and (b) infant and maternal/paternal facial expressions of emotion, which were then grouped into categories of interpersonal engagement (see Kokkinaki, 2013, 2018; Kokkinaki and Vasdekis, 2015; Kokkinaki et al., 2016 for details of the plan for microanalysis and coding).

#### Content Categories

The content of parental infant-directed speech was assessed according to the following categories: thematic sequences, non-speech sounds, vocal expressions, vocal and verbal games, and songs. A thematic sequence was defined as a block of utterances, that is, units of spoken language marked off on either side by a pause, with a high degree of semantic coherence (Kerbrat-Orecchioni, 1990; Siegel, 1963; Rabain-Jamin and Sabeau-Jouannet, 1997; Rondal, 1980; for definitions of the rest content categories see Kokkinaki, 2013). In this study, speech acts were coded according to the occurrence of questions, or directive categories, in utterances comprising the thematic sequence(s).

#### Focus Categories and Thematic Sequences

Table 2 presents the categorization of parental infant-directed speech according to focus categories and thematic sequences.

**TABLE 2:** Description of focus categories with thematic sequences of parental infant-directed speech.

*Focus Categories*	Description of Thematic Sequences
Infant-Focus	In response to, or description of infant’s internal state, external and physiological state, and infant’s body movements. Infant internal state thematic sequences included the description of attention/gaze behavior, emotion(s), communicative ability, desire(s), autonomy, character/temperament, knowledge/thought process/memory/learning, or the parent was talking from the infant’s perspective. Infant external state thematic sequences either described infant appearance or expressed admiration for it.
Dyad-focus	The parent attempts to communicate with the infant, or to describe the dyadic and bi-directional emotional/behavioral exchange and sharing of expressive behaviors (e.g., emotions, gaze direction), physiological states, body parts, appearance, and position in space.
Parent-focus	In reference to maternal/paternal behavior(s), emotion(s), and desire(s).
Other-focus	Comments on an external situation, on an object/toy, or on a third person.

#### Segmentation Procedure

Each of the above focuses and thematic sequence categories of parental infant-directed speech were measured to an accuracy of 1/25th of a second and segmented into *units* and *sub-units* of analysis. In this study, a *sub-unit* was defined as the temporal period that began at the start of one thematic sequence, or an infant vocalization to which the parent responded to, and ended at the termination of parental response to an infant cue, or at the completion of the thematic sequence. The *unit* of analysis consisted of one or more subunits depending on their between-pause duration. If the duration of pause between two successive subunits was shorter than or equal to 2 s, these were grouped within the same unit. This means that units of analysis are separated by intervals longer than 2 s. According to Herrera et al. (2004), the 2 s pause is adequate for the change of content of parental utterances.

#### Coding of Speech Acts

##### Questions

A question was defined as an utterance ending in a question mark seeking information, or confirmation (Snow, 1977; Elias et al., 1988; Pancsofar and Vernon-Feagans, 2006). Questions were grouped as follows: (1) open-ended questions, (2) close-ended questions, (3) two alternative questions, and (4) questions, including answers (for detailed definitions with examples see Kokkinaki, 2013).

##### Directives

*Direct requests* were imperatives which require the addressee to bring about a state of affairs (Levinson, 1984; Stephany and Voelkova, 2015). Coding of direct requests included: (1) direct commands, that is, utterances that controlled the physical behavior of the child by commanding him/her to do or desist from doing something; (2) attentional directives that sought to attract, direct, or redirect the child’s attention (Reissland, 1998); and (3) prompting a certain infant emotional expression.

#### Coding of Infant and Parental Facial Expressions of Emotion

Detailed analysis of timing of the types and the intensity of facial expressions of emotion was continuous in the course of each identified sub-unit of analysis and it carried out to an accuracy of 1/25th of a second. For the aim of this study, micro-analysis of infant and parental emotional expressions and intensity corresponded only to those subunits of analysis which included thematic sequence(s) of parental speech in which utterances were expressed in questions and directives. Micro-analysis of emotional expressions and intensity was carried out by the first researcher (first observer).

#### Types of Facial Expressions of Emotion

Within each identified sub-unit, the coding scheme predicted *the following types of facial expressions*: (1) *happy*, that is (a) pleasure expressed to the partner or (b) pleasure expressed to an external object/environment; (2) *interest*, which is (a) expressed to the partner or (b) expressed to an object/environment; (3) *neutral* expressions; and (4) *sad or withdrawn* expressions [see Kokkinaki (1998, 2015) for the theoretical framework].

#### Intensity of Facial Expressions of Emotion

In each identified sub-unit of analysis, the onset time in the emotional expression of one partner coincided with the offset time of the previous emotion of the same partner. When the parent addressed thematic sequences to the infant in which questions and directives were identified, it was most likely that a parent and infant would each express more than one type of facial expression of emotion. In order to obtain a full description of the change of intensity of emotional engagement over time, *the valence of facial expression* within each category of emotion was recorded by a symbol (Table 3). The sequential order of these symbols for each partner in the course of each sub-unit of analysis determined *three categories for the direction of emotional intensity change*: ascending, descending, and fluctuating.

**TABLE 3:** Definitions of the qualities of emotional valence and the categories of direction of emotional intensity change.

Qualities of emotional valence	
Positive emotional valence	(+++) pleasure to the partner, (++) pleasure to inanimate world, (+) interest directed to the partner
Neutral emotional valence	(0) neutral facial expression and interest directed to external world
Negative emotional valence	(−) negative facial expression
**Categories of direction of emotional intensity change**	
Ascending emotional intensity	The quality of emotional valence of one partner at the end of the subunit of analysis is *higher* in the scale than the emotional valence of the same partner in the beginning of the subunit of analysis [e.g., when the infant changed from neutral (0) to interest directed to the partner (+)].
Descending emotional intensity	The quality of emotional valence of one partner at the end of the subunit of analysis is *lower* than the emotional valence of the same partner in the beginning of the subunit of analysis [e.g., when neutral expression (0) was followed by negative emotional expression (−)].
Fluctuating emotional intensity	the emotional valence of one partner in the beginning and at the end of the subunit of analysis is the *same* in position in the scale while the intermediate valence(s) differ [e.g., when pleasure to the partner (+++) was followed by interest expression (+) and this was followed by pleasure (+++) again].

#### Interpersonal Engagement Categories

**DIAGRAM 1:**
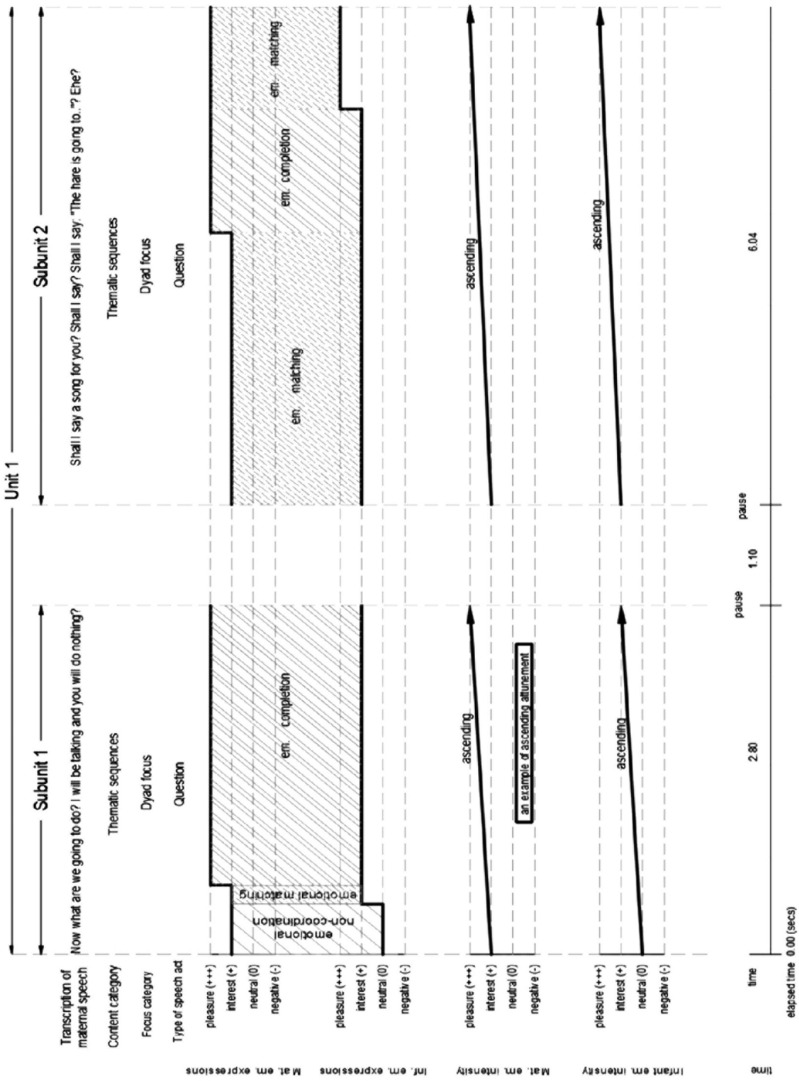
An example of micro analysis of facical expressions of emotions in the course of maternal questions in a spontaneous interaction of a 2-month-old infant with his mother.

In this study, *emotional coordination* was assessed with three categories: (a) in cases of *matching*, one partner (infant/parent) expressed the type of facial expression of emotion of the other partner; (b) in instances of *completion*, one partner expressed a positive valence of facial expression of emotion, that is, *‘pleasure’* or *‘interest,’* in immediate response to the other partner; (c) in *synchrony*, the two partners matched the timing of change of emotional expressions with each other; and (d) in *attunement*, one partner expressed the shifts in the direction of emotional intensity of the other partner. In cases of *emotional non-coordination*, the two partners did not match the type of facial expression of emotion, did not synchronize their shifts of emotions, or one partner did not attune to the direction of emotional intensity of the other partner (Kokkinaki, 2015) (see Diagram 1 for an example of micro-analysis of spontaneous infant/mother facial expressions of emotion in the course of maternal questions).

##### Inter-observer reliability

With the aim of assessing inter-observer reliability, a second observer who had been trained in the application of the coding system but was not aware of the hypotheses under investigation scored a random sample of 33% of the video files. Inter-observer reliability assessments were calculated between the coding of the first observer (researcher) and the coding of the second observer. Inter-observer reliability was carried out separately for the occurrence of questions, direct requests, the type of facial expressions, the frequency of shifts of emotion, and the categories of emotional intensity. Inter-observer reliability ranged totally from 0.75 to 0.87, with the mean value of *k* (Cohen’s kappa) being 0.80.

### Statistical Analysis

Simple comparisons of proportions between parents’ genders were accomplished using the Fisher’s exact test. Loglinear Modeling (Christensen, 1998) was used to determine relationships between variables and test hypotheses presented in the previous section. Each loglinear model was fit as a Poisson model with log-link exploring associations between triplets of variables. This was accomplished by initially fitting, for each variable triplet, the saturated model including all main effects, all two-way interactions and the three-way interaction. Then, backward selection using the likelihood ratio test (LRT) determined the optimal model. Goodness-of-fit of the finally chosen model was confirmed with a non-significant LRT test against the saturated one. The optimal model contained only statistically significant interactions. These are shown in Table 4. The significance level for goodness-of-fit tests was set at 5%. Depending on the interactions included into the final model, we quantified the association between each triplet of variable presenting appropriate proportions.

**TABLE 4:** Summary table of Model Selection Analysis; LRT tests statistical equation with Saturated Model.

Triplets of characteristics of interest	Interactions remaining in finally chosen model	LRT (*p*-value)
**Parental Questions**		
(1) Infant Em. Shifts-Parental Em. Shifts-Parental Gender	Infant Em. Shifts*Parental Em. Shifts, Parental Em. Shifts*Parental Gender	23.628 (<0.001), 6.43 (0.040)
(2) Infant Positive Em. Exp.-Parental Em. Exp.-Parental Gender	Infant Pos. Em. Exp.*Parental Em. Exp.*Parental Gender	5.203 (0.023)
(3) Infant Negative Em. Exp.-Parental Em. Exp.-Parental Gender	Infant Neg. Em. Exp *Parental Em. Exp., Parental Gender *Infant Neg. Em. Exp.	129.799 (<0.001), 45.03 (<0.001)
(4) Infant Em. Intensity- Parental Em. Intensity-Parental Gender	Infant Em. Intensity* Parental Em. I ntensity	37.76 (<0.001)
**Parental Direct Requests**		
(5) Infant Em. Shifts-Parental Em. Shifts-Parental Gender	Parental Em. Shifts*Parental Gender	11.560 (0.003)
(6) Infant Positive Em. Exp.-Parental Em. Exp.-Parental Gender	Infant Pos. Em. Exp.*Parental Em. Exp., Infant Pos. Em. Exp.* Parental Gender	8.38 (0.004), 5.44 (0.020)
(7) Infant Negative Em. Exp.-Parental Em. Exp.-Parental Gender	Infant Neg. Em. Exp *Parental Em. Exp., Parental Gender *Infant Neg. Em. Exp.	43.46 (<0.001), 10.42 (0.005)
(8) Infant Em. Intensity- Parental Em. Intensity-Parental Gender	Infant Em. Intensity* Parental Em. I ntensity,	11.83 (0.019)

As regards the effects of age, we restricted analysis for the relationship between infant age and infant–parent emotional matching and non-coordination only during questions because questions were the predominant speech act in both infant–mother [questions: 1530 (62.5%), direct requests: 916 (37.5%)] and infant–father interactions [questions: 1608 (84.5%), direct requests: 295 (15.5%)]. To take into account the different number of subunits and emotional matchings of pleasure/interest for each dyad and at each age point, we calculated a composite index. This index was defined as the division of the total number of emotional matchings of pleasure/interest in the course of maternal and paternal questions for each age specific point to the total number of emotional matchings for all the subunits (with the respective parent) for the same age point. The range of this index was expressed as a number between 0 and 1. For example, an index of 0.70 (7/10) at 2 months, corresponding to mother–infant interaction of subject n10, means that from the total number of 10 cases of emotional matchings that occurred in the course of maternal infant-directed speech of this dyad at 2 months, and seven of them were expressed during maternal infant-directed questions at the same age level (2 months). The same index was calculated for the case of emotional non-coordination in which the parent expressed positive emotion (pleasure, interest) and the infant expressed either interest to the external world or he/she was in a negative emotional state. Instances in which the parent was in a negative emotional state were not included in this analysis due to low frequencies. Then, repeated measures analysis of variance (ANOVAs) with Huynh–Feldt correction was conducted to see if there was a significant interaction effect of parent and infant age or an effect of age or parents’ gender. In case of a significant effect, multiple comparisons using Bonferroni correction provided the statistically significant differences. Polynomial contrasts on age or on the interaction of age and parents’ gender were taken into account to suggest trends of the composite index on age for possible further analysis. The significance level was set at 5%. All analyses were performed using SPSS statistical package (Version 25, 2017).

## Results

### Amount and Types of Parental Infant-Directed Questions in Spontaneous Infant–Mother and Infant–Father Interactions

We showed no difference in the amount mothers (35.9%) and fathers (36.7%) addressed questions to their infants (*p* = 0.460). According to the types of question, we found no variations in the amount mothers (29.4%) and fathers (28.8%) addressed open-ended questions (*p* = 0.523), close-ended questions (mothers: 13.5%, fathers: 14.7%) (*p* = 0.101), or alternative questions (mothers: 0.3%, fathers: 0.5%) (*p* = 0.191) to their infants. In the meanwhile, mothers addressed questions with answers (3.3%) to their infants more often compared to fathers (1.4%) (*p* < 0.001). Further, mothers and fathers differed in the number of direct requests they addressed to their infants. In connection to this, maternal infant-directed speech included direct requests (21.5%) more often than paternal speech to infants (6.7%) (*p* < 0.001).

### Emotional Engagement During Parental Questions (Hypothesis 1a)

#### Relationship Between Infant and Parental Shifts of Emotional Expressions

We showed that knowing how many times parents shifted their emotions during questions, infants changed their emotional expressions in the same way in interactions with mothers and fathers (Table 4, row 1). In particular, when parents changed their emotions once, infants synchronized the shift of their emotional expressions by changing once (44.7%) more frequently than twice (36.6%), or three times (18.7%). In cases in which parents changed their emotions twice, infants shifted similarly their emotional expressions twice (42.6%) more frequently than once (26.5%), or three times (30.9%). When parents shifted their emotions three times, it was more likely for infants to change their expressions twice (45.0%) than once (20.0%) or three times (35.0%). Further, paternal infant-directed questions are more often accompanied by one shift in emotions (52.6%) compared to maternal questions (44.9%).

#### Relationship Between Infant and Parental Emotional Expressions

Analysis indicates a different pattern in the way infants coordinated the type of their *positive* emotional expressions with mothers and fathers in the course of parental questions (row 2). When mothers expressed interest to their infant, infants expressed interest (95.5%) slightly more often than when mothers expressed pleasure to their infants (91.8%). This indicates that in the course of maternal infant-directed questions, *matching of interest expressions* occurs slightly more often—or to the same extent—*than emotional completion* (with a 3.7% variation). In cases in which fathers expressed interest in their infant, infants expressed interest (96.3%) more often than when fathers expressed pleasure to their infants (86.3%). This indicates that in the course of paternal infant-directed questions, *matching of interest expressions occurs slightly more often than emotional completion.*

Further analysis indicates that parents coordinated their emotional expressions of pleasure and interest in the same way in cases in which infants were in a negative or a neutral emotional state (row 3). In particular, when infants were in a negative emotional state, parents more often expressed interest in the infant (76%) than pleasure (24%) (with a 52% prevalence). In cases in which infants were interested in the external world, parents were more often pleased to their infants than interested in them (53.1% vs. 46.9% with a 6.2% prevalence). Taken these together, parental expressions of interest and pleasure differed according to infant negative and neutral emotions and variations between parental pleasure and interest were larger in cases of infant negative expressions than in cases of infant neutral state.

Further, in the course of parental questions, infant negative emotional expressions were more often in interactions with mothers (24.8%) than with fathers (15.4%) while the reverse was observed for infant neutral expressions which were more often in interactions with fathers (40.7%) than with mothers (31.2%).

#### Relationship Between Infant and Parental Direction of Emotional Intensity

We confirmed that the way infants coordinated the direction of emotional intensity is the same in interactions with mothers and fathers in the course of parental questions (row 4). In particular, when parental emotional expressions were ascending in intensity, infant emotional expressions were equally ascending (40.7%) and fluctuating (40.7%) than descending (18.5%). When parental expressions changed in a descending way, infants’ emotions changed more frequently in a fluctuating (39.6%) than an ascending way (21.7%), but they changed almost in the same probability in a descending way (38.7%).

### Emotional Engagement in the Course of Parental Direct Requests (Hypothesis 1b)

#### Relationship Between Infant and Parental Shifts in the Frequency of Emotional Expressions

We showed that when parents addressed direct signals to their infants, there is no relationship between parental and infant shifts of emotional expressions in interactions with mothers and fathers. Further, paternal directive requests are more often accompanied by one shift in emotions (59.6%) compared to maternal directive signals (42.3%) (row 5).

#### Relationship Between Infant and Parental Emotional Expressions

In the course of parental direct requests, we showed that knowing which type of *positive* emotion infants expressed, parental facial expressions of emotion changed in the same way in interactions with their mothers and fathers (row 6). In particular, when mothers and fathers expressed pleasure to their infant, infants were more likely to express interest (91.4%) than pleasure (8.6%). This indicates that *emotional completion* with the mother and the father is more frequent than *matching of pleasure expressions*. In cases in which mothers and fathers expressed interest in their infant, infants were more likely to share the same emotion (interest) (96%) than complete it with an emotion of similar valence (pleasure) (4.0%). This results in more *matching of interest expressions* than emotional completion with both fathers and mothers. Furthermore, in the course of parental direct requests, infants expressed interest to the father (97.2%) slightly more often than to the mother (93.3%).

Further analysis indicates that parents coordinated their emotional expressions of pleasure and interest in the same way in cases in which infants were in a negative or a neutral emotional state (row 7). In particular, when infants were in a negative emotional state, parents expressed more often interest in the infant (80.9%) than pleasure (19.1%, a 61.8% prevalence). In cases in which infants were interested in the external world, parents were more often pleased to their infants than interested in them (56.6% vs. 43.4%, a 13.2% prevalence). Taken these together, parental expressions of interest and pleasure differed according to infant negative and neutral emotions and variations between parental pleasure and interest were larger in cases of infant negative expressions than in cases of infant neutral state.

Further, in the course of parental direct requests, infant negative emotional expressions were more often in interactions with mothers (84.0%) than with fathers (16%).

#### Relationship Between Infant and Parental Direction of Emotional Intensity

We showed that the relationship between infant and parental shifts in the direction of their emotional intensity does not differ between interactions of infants with their mothers and fathers (row 8). In particular, when parents shifted their emotional expressions in an ascending way, infants’ expressions were more likely to be fluctuating (40.4%) than descending (21.1%), but they changed almost in the same probability in a ascending way (38.6%). In cases in which parental emotional expressions changed in a descending way, infants’ emotional expressions changed more often in a fluctuating (49.5%) than an ascending intensity (19.6%).

#### Relationship Between Infant Age, Parental Gender, and Infant–Parent Emotional Matching/Non-coordination in the Course of Questions (Hypothesis 2)

**FIGURE 1:**
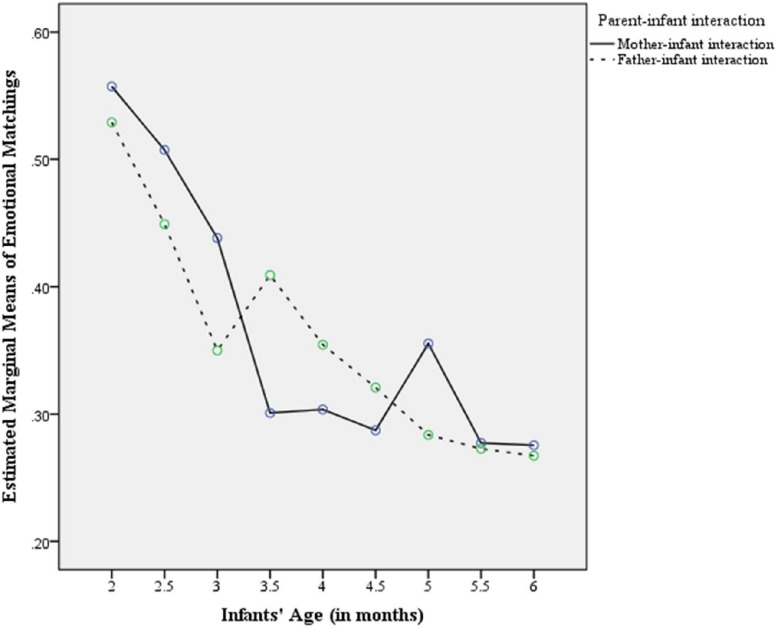
Developmental curves of pleasure and interest emotional matching in the course of parental infant-directed questions across the age range of this study (2nd to 6th month of infant’s life).

Repeated measures analysis of variance showed a significant infant age effect on emotional matchings in the course of questions, *F*(6.61,132.17) = 7.044, *p* < 0.001, the same for both parents, *F*(6.61,132.17) = 0.816, *p* = 0.583. In particular, we noticed (Figure 1) that the developmental trajectory of face-to-face emotional matchings between both mother– and father–infant interactions is descending almost linearly across the age range of this study forming a plateau at the largest age values. This was confirmed by inspection of the polynomial age contrasts which indicated a significant linear effect, *F*(1.20) = 27.667, *p* < 0.001, and quadratic effect, *F*(1.20) = 5.145, *p* = 0.047. As regards comparisons between ages, emotional matchings varied significantly between 2 and 5 months (mean difference: 0.224, standard error: 0.051) (*p* = 0.049), 2 and 5.5 months (mean difference: 0.268, standard error: 0.056) (*p* = 0.028), 2 and 6 months (mean difference: 0.272, standard error: 0.050) (*p* = 0.010), 2 and 4 months (mean difference: 0.214, standard error: 0.051) (*p* = 0.017), and 2.5 and 6 months (mean difference: 0.207, standard error: 0.054) (*p* = 0.036) (Figure 1).

**FIGURE 2:**
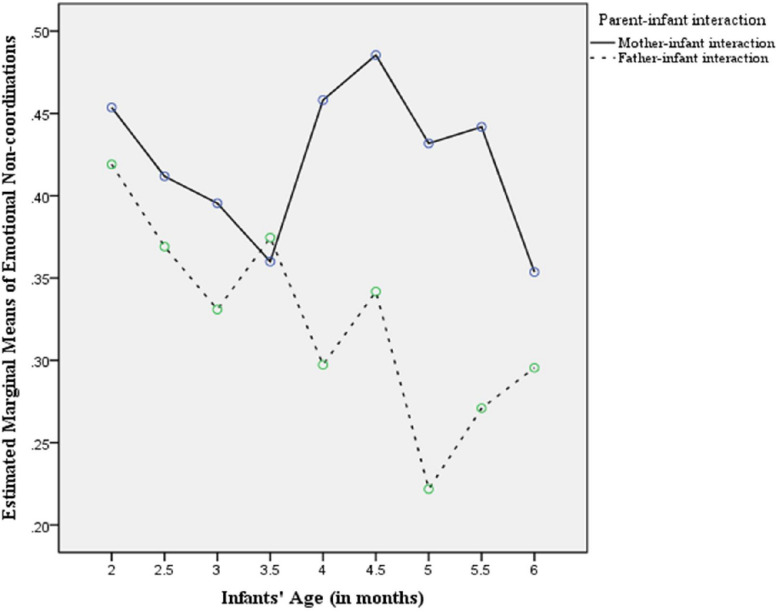
Developmental curves of emotional non-coordination in the course of parental infant-directed questions across the age range of this study (2nd to 6th month of infant’s life).

Further, analysis showed non-significant effects of (infant age)^∗^(parent gender), *F*(5.45,108.98) = 0.924, *p* = 0.477, parent gender, *F*(1.00,20.00) = 3.547, *p* = 0.089, and infant age, *F*(5.45,108.98) = 0.764, *p* = 0.588, on the developmental trajectory of emotional non-coordination in the course of parental infant-directed questions (Figure 2). Despite the non-significant effects of infant age, parent gender and (infant age)^∗^(parent gender) interaction on the developmental trajectory of emotional non-coordination, inspection of the polynomial age contrasts indicated of a negative linear trend on age different between parents’ gender, *F*(1.20) = 5.785, *p* = 0.037, with fathers showing the steepest slope in terms of age.

## Discussion

We aimed to compare the way infants unfolded their facial expressions of emotion in relation to parental expressions of feelings between maternal and paternal questions and direct requests.

### Summary of Main Results

We confirmed (Hypothesis 1a) *to a certain extent*. As is consistent with our expectations, when mothers and fathers addressed questions to their infants, we found similarities in the way infants synchronized the shifts of their emotional expressions. In addition, we showed *slight variations* between the two dyads only for the type of positive facial expressions. In cases in which infants were in a negative emotional state, both parents responded with sensitivity by expressing interest in the infant more often than pleasure. In contrast to our expectations, we showed similarities in the way infants attuned the intensity of their emotional expressions to their parents, and we found similar instances of emotional non-coordination when the infant did not attune to the direction of emotional intensity of the parent.

Similarly, we confirmed (Hypothesis 1b) *to a certain extent.* In contrast to our expectations, when mothers and fathers addressed direct requests to their infants, we showed similarities in the way infants unfolded their facial expressions of emotion to match and complete those of their mothers and fathers. In accordance with our expectations, in cases in which infants were in a negative emotional state, both parents responded with sensitivity by expressing more often interest in the infant than pleasure. In contrast to our expectations, when parents addressed direct signals to their infants, we provided evidence of similar instances of emotional non-coordination when (a) infants did not synchronize the shifts of their emotions to their parents and (b) when the infant did not attune to the direction of emotional intensity of the parent.

We confirmed (Hypothesis 2) to *a certain extent*. We showed that infant age affected significantly, and in a similar way between the two dyads, the developmental trajectories of emotional matching but not the developmental course of emotional non-coordination during maternal and paternal infant-directed questions. Regarding the development of infant–parent emotional non-coordination, we indicated a linear trend on age difference between parents’ gender with fathers showing a steepest slope on age.

Similarities in the amount of maternal and paternal infant-directed questions and the predominance of maternal over paternal direct requests to infants is in accordance with relevant studies (Kruper and Uzgiris, 1987; Laflamme et al., 2002; Kokkinaki, 2018). In addition, similarities in the way infants synchronize, match, complete and attune their emotional expressions with their mothers and fathers are in partial consistency with findings of relevant studies (Braungart-Rieker et al., 1998; Feldman, 2003; Jia, 2014; Kokkinaki and Vasdekis, 2015; Cerezo et al., 2017). The significant effect of infant age on emotional matchings in the course of questions, which is the same for both parents, is in partial consistency with relevant findings (Kokkinaki and Vasdekis, 2015).

### Discussion and Interpretation of Main Results

#### Emotional Coordination Between Infant–Mother and Infant–Father Interactions in the Course of Parental Questions (Hypothesis 1a)

We showed similarities in the way infants coordinated their emotional expressions in synchrony, matching, and attunement with mothers and fathers in the course of parental questions. In infant–caretaker intersubjective interactions, motives manifested in emotional expressions reveal coordination in three essential dimensions of communication: ‘*kinematics’* (changes of expressive behaviors in time), ‘*physiognomics’* (transformations in the form of expressive organs), and *‘energetics’* (variations in the intensity of expressions) (Trevarthen, 2012, 2017). On this ground, the results of this study confirm previous evidence that both infants and parents (mothers and fathers) are sensitive to the time, form, and intensity of their partner’s emotional expression (Trevarthen, 1993b; Kokkinaki, 2009; Kokkinaki et al., 2016).

Furthermore, this study showed similarities in the patterns of interruptions of cooperative dialogue in the course of both maternal and paternal questions. These were expressed by cases of emotional non-coordination when the infant did not attune to the direction of emotional intensity of the parent. Interruption of interpersonal communication with an infant signifies shifts in the motivation for communication, possibly toward a self-protecting agitation, distress, and call for attention to relieve some internal physiological need or for physical comfort (Trevarthen, 2005; Porges, 2010; Kokkinaki et al., 2016). In connection with this, this study showed that both mothers and fathers are similarly sensitive to signs of infant agitation, distress and call for attention. We showed that when infants were in a negative emotional state, both parents responded with serious attention to their infant than with pleasure. Furthermore, we showed that infant negative emotional expressions occurred more often in interactions with mothers than with fathers, while the reverse was observed for infant neutral expressions, which occurred more often in interactions with fathers than with mothers. It seems that mothers and fathers are effective affectionate companions in a complementary way in comforting infant self-protecting agitation (expressed either by negative or through neutral emotion of self-absorption).

#### Emotional Coordination Between Infant–Mother and Infant–Father Interactions in the Course of Parental Direct Requests (Hypothesis 1b)

We showed similar patterns of emotional coordination and non-coordination between periods in which mothers and fathers made direct requests to their infants. Interpersonal engagement profiles that accompany early parental direct requests may be precursors of the way infants’ awareness of the adult’s communicative intention emerges within a *‘response space’* created by the parents’ directives (Reddy, 2015). In connection to this, these profiles add support to the assumption that ‘…*embodied directives demonstrate how mutual accountability, and trust are produced and instantiated through family members’ moment-to-moment participation in everyday interactions*’ (Cekaite, 2010, p. 20). Directive trajectories influence the ongoing production of the intentional action (Cekaite, 2010). In particular, in the course of directive/response sequence, parental attention to the infant interest expressions (*matching of interest expressions)* may reflect the speaker’s effort (parent) to monitor the body of her addressee (infant). This will enable the speaker (parent) to assess whether his/her talk is receiving the forms of alignment and co-participation that she proposes to be relevant. The configuration of bodies into facing formations permit direct face-to-face interpersonal communication through close parent–child alignment in an intercorporeal framework for mutual engagement (Cekaite, 2010). This example may constitute an early developmental path for the embodied shape and the interactional architecture of directives in toddlers and children through which their active participation in intimate interpersonal relations is accomplished.

We provided evidence of similar instances of emotional non-coordination in the two dyads when (a) infants did not synchronize the shifts of their emotions to their parents and (b) when the infant did not attune to the direction of emotional intensity of the parent. It seems that interruptions of cooperative dialogue between parent-infant pairs vary more during parental direct requests compared to questions. On the basis of this, we assume that preverbal infants may use their affections in spontaneous interaction with their parents to express early signs of sensitivity to the different intentions inherent in parental infant-directed questions and directives. This suggestion invites further empirical investigation.

#### Relationship Between Infant Age, Parental Gender, and Infant–Parent Emotional Matching/Non-coordination in the Course of Questions (Hypothesis 2)

This study indicated that the developmental trajectory of face-to-face emotional matching in the course of questions follows a similar decreasing trend in interactions of infants with their mothers and fathers across the age range of this study (2nd to 6th month of life). This is in accordance with developments in the body and in the motivating processes of the infant brain. These developments trigger transformations in the way infants regulate their feelings and express their interests to their companions at certain ages (Trevarthen, 2011). After 6 weeks, in the period of ‘*Primary Intersubjectivity,’* during intimate protoconversations, the young infant shows direct sensitivity to the timing and values of facial expressions of feeling in intimate contact with caregivers (Trevarthen, 1993b). From 3 to 6 months of life, in the ‘*Period of Games,’* the infant becomes more playful, responding to rhythmic provocations of expressive movements in ‘person–person games’ and body play. As a consequence of this transformation, infants lose their excitement for face-to-face proto-conversation while exploration of the environment increases and the manipulation of objects becomes effective (Trevarthen, 2017).

Further, we showed non-significant effects of infant age, parent gender and (infant age)^∗^(parent gender) interaction on the developmental trajectory of emotional non-coordination. Despite these non-significant effects, we showed a negative linear trend on age different between interaction of infants with mothers and fathers, with fathers showing a steepest slope on age (Figure 2). This finding does not verify our expectations of an upward trend of infant-parent emotional non-coordination after the 3rd month (‘Period of Games’) due to transformations in the way infants regulate their feelings while exploring the environment. This unexpected result may be due to the following factors: (a) repeated measures analysis of variance was based on a composite index which incorporated both cases of infant external interest expressions and infant negative emotional states (see in the section of Statistical Analysis), and the inclusion of infant negative emotions in this case may thus restrict us from getting a full picture of the transformations in the way infants regulate their emotional expressions and explore the environment after the 3rd month; (b) we did not include in this analysis cases of mutual infant–parent external interest expressions since these were expressed in low frequencies by parents; and (c) the developmental trajectories that showed cases of infant–parent emotional non-coordination are restricted only to maternal and paternal infant-directed questions. These issues invite further research which will separate cases of infant external interest expressions from instances of infant negative emotional states. In addition, future research may include more speech acts and verbal expressions of parental infant-directed speech. This will enable us to gain a complete picture of the way parents and their infants express their emotional ‘disagreement’ on the basis of embodied intersubjectivity.

Taken these together, the developmental trajectories of infant–mother and infant–father emotional coordination in the course of parental infant-directed questions across the age range of this study imply that mothers and fathers respond with similar sensitivity to clearly marked advances in the infant’s attentiveness and playfulness, self-awareness, and engagements with objects (Trevarthen and Aitken, 2003). Further research is needed to complete the evidence of this study on the developmental course of infant–parent emotional non-coordination in the course of parental infant-directed speech.

### Potential Clinical Implications

Similarities in the way infants’ facial “unfolded” in relation to parental expressions of feelings provided evidence of the sensitive and complementary company and care both parents give to developing infant. The potential implications of this study are related to the following issues:

(a) the inclusion of both mothers and fathers in designing interventions for the promotion of early interactions and play between children with their parents. The evidence of our study may extend our understanding of the moderating role that fathers may have on the family process and on infant development (Vakrat et al., 2018); and (b) the connection between early patterns of infant-parent emotional communication with later cognitive and behavioral child development outcomes and attachment. In particular, typical development (vs. psychopathological development), and more positive child outcomes have been correlated with better mother–child synchrony. The quality of social interactions is a function of an active infant–parent ‘dialogue’ and depends on the infant’s desire to be interactive and the parent’s capacity to be attuned. Mother–child interactions are poorer in synchrony when one of the partners is impeded by internal (pathological) or environmental distractor. For instance, on the one hand, studies with depressed mothers (with limited maternal sensitivity and empathy) showed a trend to less synchrony/coherence. On the other hand, interactions of mothers with their premature infants showed lower coherence. Regarding environmental distractors, among high-risk, low-income toddler boys, synchrony was negatively associated with child emotional negativity (for a full discussion see Leclère et al., 2014).

In the meanwhile, during the first year of life, “Synchrony is not an all or nothing concept; rather, it may be more valid to think about dyadic interactions as approaching or moving away from synchrony” (p. 23, Leclère et al., 2014). On this ground, fluctuations in infant–parent emotional regulation, which imply both breaks of communication along with short ‘real’ parent–infant dialogues^1^, are important for the promotion of adaptation, creativity and stimulation (Leclère et al., 2014; Kokkinaki et al., 2016). The findings of this study may thus add understanding for the developmental importance of interactive errors and repairs in early infancy (Tronick and Cohn, 1989). Further research will inform us on the long-term implications of these engagement patterns on child development outcomes.

### Limitations of This Study

The conclusions of this study are restricted in certain ways. The number of subjects was small to allow for efficient measurement of the between-subjects variability. Coherence in interaction patterns of mothers and fathers with their infants as a consequence of their marital life and caregiving may prevent us from identifying the possible differences between them (Cerezo et al., 2017). Our conclusions come from a Cretan (Greek) population, and they need to be verified through replication of the study in other cultures with different beliefs of how parents should give company and care to their developing infant. The families of infants chosen for this study are not representative of the whole population, being families who volunteered. From the current study, no inferences can be made with respect to causality.

Although we video recorded infant–parent pairs in a naturalistic environment familiar to participants, asking parents to play with their baby while being video recorded by the researcher may have been felt to be intrusive. Despite the limited knowledge on this matter, the gender of the parent may influence reactivity (Gardner, 2000). Mothers express a higher frequency of behaviors in situations of video recorded play than during the everyday observations (Abels et al., 2017). The natural behavior of fathers may have been more influenced than mothers by observation (Russell et al., 1992). Furthermore, data coming from language input in the course of naturalistic observations, showing striking fluctuations interspersed with silence, may provide a different picture of infant language experiences than that of structured play, which is consistently dense (Tamis-LeMonda et al., 2017).

## Conclusion

In sum, we have provided evidence both maternal and paternal infant-directed questions along with direct requests constitute examples of early embodied intersubjectivity. Here, intersubjectivity is expressed through facial expressions of emotional coordination and non-coordination as well as through age-related changes of interpersonal engagement. These changes reflect transformations in the way infants regulate their feelings and express their interests to their companions in the first semester of life. Further, similarities in the way infants ‘*unfolded’* their facial expressions of emotion in relation to parental expressions of feelings between maternal and paternal questions and direct requests, may imply that the human infant is born ready to actively participate in short ‘real’ dialogues with both the mother and the father. We provided preliminary evidence that preverbal infants may use their affections in spontaneous interaction with their parents to express early signs of sensitivity to the different intentions inherent in parental infant-directed questions and directives. This invites further empirical investigation. Additionally, parents, both the mother and the father, are sensitive to the infant’s capacities for emotional regulation, coordination, and communication of feelings (Trevarthen et al., 2006; Trevarthen, 2011, 2012, 2017).

## Author’s Note

The video recordings of spontaneous infant–parent interactions used in this study were made for the Ph.D. research of the first author at the Department of Psychology, University of Edinburgh, under the supervision of Prof. Colwyn Trevarthen.

## Data Availability Statement

The raw data are not publicly available due to privacy or ethical restrictions.

## Ethics Statement

The studies involving human participants were reviewed and approved by the Lothian Research Ethics Committee and the Psychiatry/Clinical Psychology Research Ethics Sub-Committee of the Royal Infirmary of Edinburgh (NHS Trust). Written informed consent to participate in this study was provided by the participants’ legal guardian/next of kin.

## Author Contributions

TK carried out this research and authored the sections “Introduction,” “Materials and Methods,” and “Discussion.” VV authored the sections “Statistical Analysis” and “Results”. Both authors contributed to manuscript revision and approved the submitted version.

## Conflict of Interest

The authors declare that the research was conducted in the absence of any commercial or financial relationships that could be construed as a potential conflict of interest.

## Footnotes

1 We thank reviewer 1 for the suggestion of the phrase “short ‘real’ dialogues,” we adopted.
